# Stability and change in the distribution of cytospecies of the *Simulium damnosum* complex (Diptera: Simuliidae) in southern Ghana from 1971 to 2011

**DOI:** 10.1186/1756-3305-6-205

**Published:** 2013-07-13

**Authors:** Rory J Post, Robert A Cheke, Daniel A Boakye, Michael D Wilson, Mike Y Osei-Atweneboana, Anthony Tetteh-Kumah, Poppy HL Lamberton, J Lee Crainey, Laurent Yaméogo, María-Gloria Basáñez

**Affiliations:** 1 School of Natural Sciences and Psychology, Liverpool John Moores University, Byrom Street, Liverpool L3 3AH, UK; 2 Disease Control Department, London School of Hygiene & Tropical Medicine, Keppel Street, WC1E 7HT, London, UK; 3 Natural Resources Institute, University of Greenwich at Medway, Central Avenue, Kent, Chatham Maritime ME4 4TB, UK; 4 Department of Infectious Disease Epidemiology, School of Public Health, Faculty of Medicine, Imperial College London, St Mary’s Campus, W2 1PG, London, Norfolk Place, UK; 5 Noguchi Memorial Institute for Medical Research, University of Ghana, PO Box LG581, Legon, Accra, Ghana; 6 Water Research Institute, Council for Scientific & Industrial Research, PO Box M32, Accra, Ghana; 7 Ghana Health Service, Private Mail Bag, Ministries, Accra, Ghana; 8 Instituto Leonidas y Maria Deane, Fundação Oswaldo Cruz, Rua Terezina 476, Adrianopolis, AM 69057–070, Manaus, Brazil; 9 African Programme for Onchocerciasis Control, World Health Organization, BP 549, Ouagadougou 01, Burkina Faso

**Keywords:** Ghana, Onchocerciasis, *Simulium damnosum*, *Simulium sirbanum*, *Simulium squamosum*, *Simulium yahense*, *Simulium sanctipauli*, *Simulium soubrense*, Cytospecies distribution, Stability, Change

## Abstract

**Background:**

*Simulium damnosum* s.l., the most important vector of onchocerciasis in Africa, is a complex of sibling species that have been described on the basis of differences in their larval polytene chromosomes. These (cyto) species differ in their geographical distributions, ecologies and epidemiological roles. In Ghana, distributional changes have been recorded as a consequence of vector control and environmental change (e.g. deforestation), with potential disease consequences. We review the distribution of cytospecies in southern Ghana and report changes observed with reference to historical data collated from 1971 to 2005 and new identifications made between 2006 and 2011.

**Methods/Results:**

Larvae were collected from riverine breeding sites, fixed in Carnoy’s solution and chromosome preparations made. Cytotaxonomic identifications from 1,232 samples (including 49 new samples) were analysed. We report long-term stability in cytospecies distribution in the rivers Afram, Akrum, Pawnpawn and Pru. For the rivers Oda, Ofin and Tano we describe (for the first time) patterns of distribution. We could not detect cytospecies composition changes in the upper Pra, and the lower Pra seems to have been stable. The elimination of the Djodji form of *S*. *sanctipauli* in the Volta Region seems to have had no long-term effects on the distribution of the other cytospecies, despite an initial surge by *S*. *yahense*. There has been a recent increase in the occurrence of savannah cytospecies in the river Asukawkaw, and this might be related to continuing deforestation.

**Conclusions:**

Cytospecies’ distributions have not been stable from 1971 to 2011. Although there are no obvious causes for the temporary appearance and subsequent disappearance of cytospecies in a particular location, a major influence has been vector control and migration patterns, probably explaining observed changes on the Black Volta and lower Volta rivers. Deforestation was previously implicated in an increase of savannah cytospecies in southern Ghana (1975–1997). Our data had little power to support (or refute) suggestions of a continuing increase, except in the Asukawkaw river basin.

## Background

For more than eighty years it has been known that the blackfly *Simulium damnosum* Theobald sensu lato (s.l.) (Diptera: Simuliidae) is the main vector in Africa of *Onchocerca volvulus* Leuckart, the causal agent of human onchocerciasis (river blindness) [1]. Hence, the geographical distribution of the disease is determined by the geographical distribution of its vector. Vector surveys in West Africa (including Ghana) up to 1968 have been listed [2], but by 1966 it had already become clear that the morphotype then known as *S*. *damnosum* was not a single species, but rather a complex of sibling species which were morphologically very similar or identical, although they could be separated on the basis of differences in their larval polytene chromosomes [3]. Early studies in West Africa revealed up to eight cytoforms [4-6], with the various sibling species showing differences in their geographical distribution and in their roles as vectors of onchocerciasis [7-9]. Many subsequent studies have broadened the range of known differences between the sibling species, which affect their importance in the transmission of onchocerciasis [10-12]. The World Health Organization (WHO) Onchocerciasis Control Programme in West Africa (OCP) was established in 1974 to control blinding savannah onchocerciasis, transmitted by the savannah cytospecies *S*. *damnosum* sensu stricto (s.str.) and *S*. *sirbanum*, using weekly aerial application of larvicide to the riverine breeding sites [13]. Cytotaxonomic identification of blackfly larvae was an indispensable tool for onchocerciasis control by larviciding [10], but subsequently this type of information was considered of only minor importance to control onchocerciasis by community-directed treatment with ivermectin (CDTI). However, the recent strategic shift from control to elimination has changed this perception, and the strategy developed by the WHO African Programme for Onchocerciasis Control (APOC) for verification of elimination includes entomological evaluation with regular collection of cytotaxonomic data in all foci [14].

Cytospecies identifications from Ghana were first published for larvae collected from five sites in July and October 1971 [5], including the Volta river in the south of the country ‘near Akosombo Dam’ (presumably Senchi Rapids). Further data were rapidly accumulated until the basic pattern of cytospecies’ distributions was ascertained in Ghana according to habitats and latitudes [15], and was found to be similar to that already described from Côte d’Ivoire [7]. Six cytospecies were recognised in Ghana. *Simulium damnosum* s.str. and *S*. *sirbanum* were widely distributed in the north of the country, and *S*. *sanctipauli* and *S*. *yahense* were recorded in the southern, forested areas (including the montane forest that straddles the border between Ghana and Togo). *Simulium soubrense* and *S*. *squamosum* seemed to be scattered around the forest/savannah mosaic.

Subsequent studies have not only provided additional information on the distribution of the cytospecies in Ghana, but they have also revealed the existence of further cytotaxonomic entities and new diagnostic criteria. Initially, identifications of *S*. *soubrense* and *S*. *sanctipauli* larvae were made using inversion 2 L-7 [5], but following criticism of this criterion [6], the diagnostic character was changed in 1986 to inversion 2 L-A [16]. (This means that identifications of *S*. *soubrense* and *S*. *sanctipauli* prior to 1986 cannot necessarily be considered reliable.) When the Beffa form was described [17], it was not possible to allocate it to either *S*. *sanctipauli* or *S*. *soubrense* because of the inadequacies of inversion 2 L-7 for species-specific diagnosis, but application of inversion 2 L-A clearly indicated that the Beffa form was a taxonomic subdivision within *S*. *soubrense*[16,18].

A common problem in the ability to interpret historical cytotaxonomic data has been the subsequent recognition of new cytotypes within previously known entities. For example, over the years *S*. *sanctipauli* has been subdivided, at first by the recognition of the Djodji form as distinct from the typical form [19,20], and later by subdivision of the typical form into the Pra and the Comoé forms in Ghana [21]. *Simulium sudanense* had been recorded in Ghana [5], but its species status was not accepted by all authors, and it was not further recognised in Ghana until 1992 when it was itself divided into two cytotypes [22]. Three cytotypes are currently recognised within *S*. *sirbanum* s.l. (sirba form, sudanense form and Type IV form) [23]. When it was first described, *S*. *squamosum* was understood to be a chromosomally variable species [5], and it is now subdivided into at least five cytotypes in West Africa [18,22], with two forms reported from Ghana [18]. These forms differ in their sex chromosomes and are known as *S*. *squamosum* C and *S*. *squamosum* E (= *S*. *squamosum* type III of [18]). Post *et al*. [22] listed *S*. *squamosum* E from Sierra Leone, Guinea, Liberia, Côte d’Ivoire, Ghana, Togo, Benin and Central African Republic, and *S*. *squamosum* C from only Cameroon and Nigeria. However, this was erroneous regarding Ghana, Togo, Benin and Central African Republic (mistakes repeated by Adler *et al*. [11]) because *S*. *squamosum* C has been identified in all of these countries, but *S*. *squamosum* E is not known from Togo, Benin and Central African Republic. In summary, *S*. *squamosum* E has been correctly identified from Sierra Leone, Guinea, Liberia, Côte d’Ivoire and Ghana, and *S*. *squamosum* C from Ghana, Togo, Benin, Nigeria, Cameroon and Central African Republic.

Wilson *et al*. [24] analysed the distribution of cytospecies in southern Ghana (and southwest Togo) and showed that there had been an increase in the frequency of savannah cytospecies during the study period (1975–1997), relating this trend to anthropogenic deforestation. Since then deforestation has continued (Table 1). If this trend continued to be associated with changes in cytospecies’ distributions, it would have important epidemiological implications, as it is the savannah cytospecies that are considered to be mostly associated with transmission of blinding onchocerciasis (but see [25]). We have, therefore, updated (to 2011) the same database of cytospecies identifications used in [24] and added some previously missing samples. The purpose of this paper is to review the distribution of the vector cytospecies of the *S*. *damnosum* complex in southern Ghana, to highlight the various changes which have been observed with reference to historical data (1971–2005) and new identifications (2006–2011) in total covering over 40 years, and to discuss the epidemiological implications of our findings.

**Table 1:** Changes in Forest Cover in Ghana over 20 years 1990-2010*

**Year of survey**	**1990**	**2000**	**2005**	**2010**
Forest area (ha)	7,447,854	6,093,906	5,516,932	4,939,958

*Data from [26].

## Methods

### Study area

**Figure 1:**
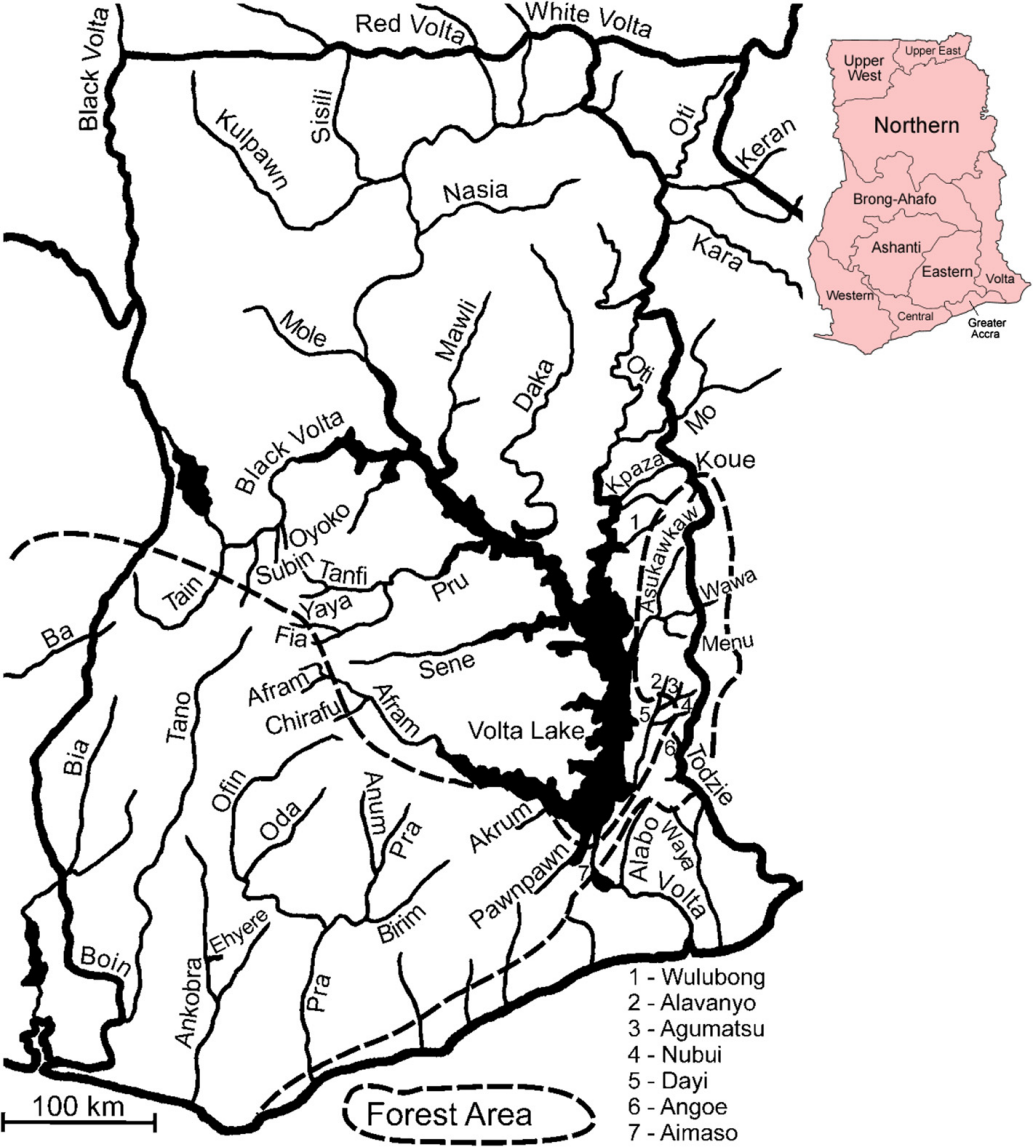
**Map of Ghana showing rivers mentioned in the text and the extent of the forest zone (inset shows Ghana regions, **http://en.wikipedia.org/wiki/File:Ghana_regions_named.png**).**

For the purposes of this paper we have defined southern Ghana as consisting of Brong Ahafo, Ashanti, Western, Central, Eastern, Greater Accra and Volta administrative regions (see inset of Figure 1). This area occupies most of Ghana south of latitude 8°47’N, it includes all the bioclimatic forest areas, and all the rivers (or their tributaries) run through forest for at least part of their length.

### Chromosomal preparations

Larvae of the *S*. *damnosum* complex were collected from their riverine breeding sites in Ghana (Figure 1) and preserved in Carnoy’s solution (3 parts absolute alcohol: 1 part glacial acetic acid). Stained preparations of the polytene chromosomes from the larval silk glands (= salivary glands) were made on microscope slides, and they were scored for species-diagnostic inversions according to standard methods available at the time (see above and [22]).

### Sources of information and database construction

Historical data from Ghana were compiled from: a) a review of the published and grey literature (various authors in various journals and unpublished reports from 1975 to 2012, based on the expert knowledge of R.J.P., R.A.C., D.A.B., M.D.W., and M.Y.O.-A. of such literature); b) the database originally compiled under the direction of D.A.B. for the OCP of all identifications it ever commissioned; c) unpublished OCP reports (authored by C.G. Vajime, 1975–1980; S.E.O. Meredith, 1980–1983; A. Weber, 1986, and G.K. Fiasorgbor, 1981–1983); d) unpublished results from authors of this paper (R.J.P., D.A.B. and M.Y.O.-A., 1980–2005), and e) new results of cytotaxonomic identifications made by authors of this paper (R.J.P., D.A.B. and M.Y.O.-A., 2006–2011). This database was previously compiled by Wilson *et al*. [24], but it has been updated with new samples (1997–2011) and previously missing samples have been added from historical sources. In building the database we cannot guarantee that we have found every published record, though we think this is likely to be the case. We have also tried to include results from all unpublished sources (such as the unpublished OCP reports), but we are aware that some of this documentation may be unavailable and/or missing. In the unlikely event that we have missed a few records, we doubt they would add anything significant to the patterns we describe.

## Results

All historical and recent results were compiled into a database file organised alphabetically by river (see Additional file 1: Cytotaxonomic identifications of the *Simulium damnosum* complex from Ghana). The date notation we use below and in Additional file 1 follows the day-month-year format, with month in roman numerals.

### Lower Volta river

The large rapids on the lower Volta river, where it passes through the Togo-Atakora mountains (the southern part of the Akwapim-Togo range) in southern Ghana, used to harbour enormous populations of *S*. *damnosum* s.l. From 1962 onwards, these populations were subject to intermittent control, using larvicidal insecticides applied from boats, by the Volta River Authority to protect the work force constructing the Akosombo Dam from the biting nuisance. The dam was completed in 1965 and the Volta lake flooded the breeding sites upstream, leaving only the Senchi and Kpong rapids downstream. Intermittent control continued to protect the local inhabitants when populations of *S*. *damnosum* s.l. built up to a level that caused complaints and when the Volta River Authority could find larvae in the rapids [27]. On each occasion control operations would be carried out for a number of weeks until biting flies and larvae had disappeared, and control would then be halted until numbers had built up again. It was reported that the recrudescent species was sometimes different from the one that had been present when control operations commenced ([27], J.N. Raybould, pers. comm., 1980). In 1981, a second dam was finished at Kpong, downstream of Akosombo, flooding the remaining two rapids (Senchi and Kpong rapids).

**Figure 2:**
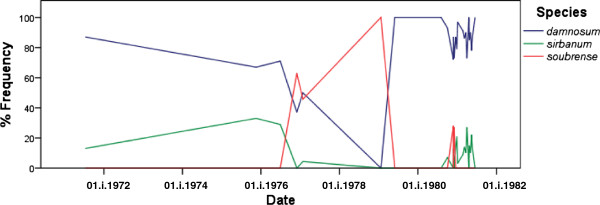
Proportions of cytospecies from Lower Volta river near Akosombo 1971–1981.

Figure 2 shows the proportions of the different cytospecies identified from Senchi Rapids during the period for which cytotaxonomic identifications are available (1971 to 1981). Both savannah cytospecies (*S*. *damnosum* s.str. and *S*. *sirbanum*) were identified together in the majority of samples throughout this period, and sometimes they were the only species present when control started and they were also the recrudescent species. For example, *S*. *damnosum* s.str. was the only species recorded in February–March 1980 and June–August 1980 (Figure 2; Additional file 1), respectively before and after control operations in April 1980 (J.N. Raybould, pers. comm., 1980). It is of course unclear whether resurgent species have reinvaded from elsewhere or whether they have survived control operations with continued breeding in the Senchi and/or Kpong rapids, or in the smaller streams in the area, which sometimes also harboured populations of the *S*. *damnosum* complex. The Aimaso stream and Alabomi stream (=Abomi stream) discharge into the Volta river near Akosombo, and *S*. *damnosum* s.str., *S*. *sirbanum* and the *S*. *sanctipauli* subcomplex have all been identified from them, at times correlated with the cytospecies present in the Volta river. These two streams, along with the rivers Akrum and Pawnpawn (less than 50 km west or west–north–west from Akosombo) also harbour *S*. *squamosum* and *S*. *yahense*, but these two species have never been identified from the lower Volta river. *Simulium sirbanum* was absent from January 1979 to October 1980, when it re-colonised the river.

Members of the *S*. *sanctipauli* subcomplex were sometimes present, possibly in relation to control operations. At the time, the identification of *S*. *sanctipauli* and *S*. *soubrense* was based on the old 2 L-7 classification, but the significant numbers of 2 L-7 heterozygotes (6%) in the sample from 26-xi-1980 [27] is a characteristic of the Beffa form of *S*. *soubrense*. It is not known whether the diagnostic inversion 2S-6b, which was not formally described until 1983 [17], was also present nor is it known who carried out the identifications listed in [27]. Meredith *et al*. [28] also listed these identifications, but did not state if they were Beffa or not. The only potential sources of reinvasion by members of the *S*. *sanctipauli* subcomplex are to the east and to the west. The nearest are the very large breeding populations southwest to west (i.e. upwind) of Akosombo on the rivers Anum and Pra. These, however, are populations of the Pra form of *S*. *sanctipauli*, which have inversion 2 L-7 almost fixed (0.95–1.00) ([21], and R. J. Post unpublished data), whereas the sample from Senchi rapids on 26-xi-1980 had a much lower frequency (0.32) [27], although this is still within the range recorded for the Beffa form of *S*. *soubrense*[17].

The adult males of the Beffa form of *S*. *soubrense* are characterised by type IV scutal patterns and this has been the only pattern reported from the *S*. *sanctipauli* subcomplex from the Volta river below Akosombo [17,29], but has not been recorded from the river Pra. It is, therefore, very likely that the member of the *S*. *sanctipauli* subcomplex recorded occasionally during the period of intermittent vector control on the Senchi and Kpong rapids was the Beffa form of *S*. *soubrense* on all, or at least some, occasions. These temporary populations would represent the most westerly point in the distribution of the Beffa form. It is interesting to note that although the Beffa form extends into the forested area of southwest Nigeria [30], it has been most commonly identified from Togo and Benin in the Dahomey Gap (where there is a gap in the rain forest belt and the savannah extends southwards to the coast). The Kpong and Senchi rapids are situated at the northern edge of the Accra Plains, which is a westward extension of the Dahomey Gap savannah along the coast of Ghana. The nearest known potential immigration source is the river Sio in Togo, approximately 100 km northeast of Akosombo, and well within the flight range estimated for Beffa form (<125 km, see Figure four of Garms *et al*. [31]).

### Rivers of southern Ghana originating from the Southern Voltaian Plateau

One of the major topographical features of southern Ghana is the Southern Voltaian Plateau, which is a forested ridge of sandstone running southeast to northwest forming the watershed which separates the rivers that run southwards directly into the sea, from the rivers which run northwards or northeastwards into the Volta river basin to join the Volta river on its way to the sea (Figure 1). The rivers Afram, Akrum and Pawnpawn (with its tributary the river Chirimfa) flow northeast from the Southern Voltaian Plateau into the Volta lake. The rivers Akrum and Pawnpawn run through forest and the river Afram runs from the forest into the Guinea savannah before discharging into the Volta lake. They are all characterised by stable populations of *S*. *squamosum* (identified as *S*. *squamosum* E from the river Pawnpawn) with a few *S*. *yahense*. We have no new samples from the rivers Afram, Akrum and Chirimfa, but six new samples from the river Pawnpawn were consistent with older data in that they consisted entirely of *S*. *squamosum* and *S*. *yahense*. The headwaters of the river Pru and its tributaries (rivers Fia, Tanfi and Yaya) also arise in the forested Southern Voltaian Plateau, but much of the Pru basin is Guinea savannah. In the past, the rivers Fia, Tanfi and Yaya have harboured populations of *S*. *squamosum* with a few *S*. *yahense*, but we have no new samples to determine whether these cytospecies have continued up to the present. All historical samples from the river Pru (mostly from Asubende) have consisted of *S*. *damnosum* s.str. and *S*. *sirbanum*, and we have a single new sample (from Asubende 07-ii-2011) which was the same.

### The Black Volta river

The Black Volta river arises in Burkina Faso and runs south to form the international border between northern Côte d’Ivoire and Ghana. It enters Ghana in the Guinea savannah zone, flows through Bui Gorge (the site of Bui Dam) and eventually into the Volta lake. However, a series of tributaries (rivers Pumpum or Kintampo, Oyoko and Subin) arise in the Southern Voltaian Plateau and flow north to join the Black Volta. These rivers are characterised by historical populations of *S*. *squamosum* with some savannah cytospecies (mostly *S*. *damnosum* s.str.). We have no new samples to see if these populations have remained stable. The river Black Volta has been characterised by large historical populations (1974–1984) identified as *S*. *soubrense*, *S*. *damnosum* s.str. and *S*. *sirbanum*, along with a single *S*. *squamosum* larva. There have been two new samples (from February 2010 and August 2011), which contained only *S*. *damnosum* s.str. and *S*. *sirbanum*. Considering all samples from all sites along the Black Volta there is a clear seasonal pattern. The area has a “modified tropical continental climate” [32] with a rainy season from May to October and a dry season November to April. Savannah flies (*S*. *damnosum* s.str. and *S*. *sirbanum*) were found in 25% and 62% of dry and wet season samples respectively, in comparison with 86% and 48% respectively for *S*. *soubrense*. Normally we would expect a northward movement of forest flies (such as *S*. *soubrense*) in the rainy season, but the Black Volta seems to show the reverse pattern. The explanation is unclear, but it is important to note that all the identifications of *S*. *soubrense* would have been based upon inversion 2 L-7 (not 2 L-A) and it is likely that they were all mistaken for *S*. *sanctipauli*, because it has been possible to recover the 2 L-A karyotype in a number of cases. Meredith *et al*. [28] published the 2 L-A status for the sample of 78 larvae collected from Agborlekame on 09-i-1982, and they were all homozygotes for 2 L-A, and hence they were *S*. *sanctipauli*, not *S*. *soubrense* (albeit *S*. *sanctipauli* without 2 L-7). Voucher specimens had been kept from some other samples and these were re-examined by G.K. Fiasorgbor and RJP and all vouchers (one larva dated 13-i-1982, one dated 20-i-1983 and three dated 09-iii-1983, all from Agborlekame) were *S*. *sanctipauli* not *S*. *soubrense*, and all these specimens were from temephos-resistant populations.

Temephos resistance first appeared in *S*. *sanctipauli* in 1980 (on the lower Bandama river in Côte d’Ivoire) [33], and six months later (by January 1981) resistant populations had reached the Black Volta river [28]. The population recorded as *S*. *soubrense* from historical identifications (1981–1984) was undoubtedly temephos-resistant *S*. *sanctipauli* (probably the Comoé form of *S*. *sanctipauli* which has a low frequency of 2 L-7 [21]). It is unclear whether the Black Volta is within the normal distribution of *S*. *sanctipauli*, or whether this was a range extension facilitated by insecticide resistance. There are only two samples identified from the Black Volta river since 1984 (February 2010 and August 2011–some time after the completion of larviciding operations in the area) and no *S*. *sanctipauli* were identified. There had been only four samples identified prior to 1981 and they were all collected in December 1974 from four different sites. All four samples contained savannah cytospecies, but 16 larvae of *S*. *soubrense* were also identified (using 2 L-7) from one sample (constituting 3% of the four collections combined, and suggesting that the Black Volta might be within the natural distribution range). In summary, it seems likely that the Black Volta harbours populations of savannah cytospecies and probably a member of the *S*. *sanctipauli* subcomplex, but this latter species remains to be confirmed since the discontinuation of larviciding.

### The Tano river

The river Tano arises in the Southern Voltaian Plateau and flows south directly into the sea. Near its source it mostly harbours populations of *S*. *yahense* and *S*. *squamosum*, with a few *S*. *damnosum* s.str. and *S*. *sanctipauli*. South of Acherensua the river flows out of the montane forest (*Antiaris*–*Chlorophora* association) into a lowland forest area (*Celtis*–*Triplochiton* association) and savannah flies become more common, but it is dominated by populations of *S*. *sanctipauli* (with a few *S*. *soubrense*). There have been no new samples since 1997. A few km east, the river Ehyere is a small tributary of the river Ankobra which has been recorded to harbour *S*. *yahense* (and a few *S*. *sanctipauli*), but there have been no recent samples.

### The Pra river

The river Pra along with its tributaries (rivers Anum, Birim, Oda and Ofin) arises on the Southern Voltaian Plateau, and like the river Tano, flows through forest to the sea. We have 21 samples covering more than 20 years (1989–2011) from the river Anum, including nine recent samples (2006–2011). *Simulium sanctipauli* was identified from most samples along with savannah cytospecies and some *S*. *yahense*. However, no significant increase (or decrease) in the frequency of the savannah cytospecies could be detected at Gyankobaa (=Yankoba) (Mann-Kendall test for trend), either taking into account all samples (Kendall score *S* = −30, *P*-value > 0.05), or only those samples collected in the second and fourth quarters of the year (when Wilson *et al*. [24] recorded changes across southern Ghana during the period 1975–1997) (*S* = −4, *P*-value > 0.10). This is probably not surprising considering the limitations of the dataset, which would probably only have had the power to detect quite large increases (see Discussion section below). However, it is also possible that the changing patterns between 1989 and 1997 detected by Wilson *et al*. [24] may have subsequently stabilised. There has only been a single sample identified from the river Birim, and there have been no new samples from the rivers Oda and Ofin since 1996. The Oda river shows a zonation, with *S*. *yahense* and *S*. *squamosum* in its headwaters, and *S*. *damnosum* s.str. downstream. The river Ofin also shows a zonation, but in its headwaters *S*. *yahense* occurs with *S*. *damnosum* s.str. and downstream, *S*. *sanctipauli* dominates with a few *S*. *damnosum* s.str. In contrast, in the upper reaches of the river Pra (above the junction with the Ofin river), *S*. *damnosum* s.str. is the dominant species, although *S*. *sanctipauli* was also found in most samples. In both the river Ofin and the river Pra (above their confluence) there has been an insufficient series of samples to determine whether these patterns have changed over the years. In the lower Pra (below its confluence with the Ofin), savannah flies occur regularly in the northern reaches, but there has been an insufficient series of samples to determine whether there have been any changes, except possibly downstream at Hemang. There have been 34 samples (1978–1995) from Hemang and savannah cytospecies were only recorded in three samples, which were in the 1980s, and there are no recent samples to see if savannah cytospecies have increased in frequency since 1997. Savannah cytospecies have never (including recent samples) been identified from the lowest rapids at Bosomase/Daboase about 10 km before the river enters the sea.

### Volta Region

*Simulium damnosum* s.str., *S*. *sirbanum*, *S*. *squamosum*, *S*. *yahense*, *S*. *sanctipauli* and *S*. *soubrense* have been listed from the part of Ghana lying east of the Volta lake (i.e. Volta Region) since the 1970s [15]. All identifications of *S*. *squamosum* C have been from east of the Volta lake (whereas *S*. *squamosum* E has only been identified from west of the Volta lake). Pre-1986 identifications of *S*. *sanctipauli* and *S*. *soubrense* from Volta Region can sometimes be interpreted reliably, despite the original identification using the old inversion 2 L-7, because in a few cases the inversion 2 L-A was also scored and in others the 2 L-7 karyotype is expected to be closely correlated with the diagnostic inversion 2 L-A. This is because the cytotypes of *S*. *sanctipauli* found around this part of Ghana (Djodji form and potentially Pra form—see below) are almost fixed 2 L-7/7 (which is not the case everywhere in Ghana), and the cytotype of *S*. *soubrense* found in Volta region and Togo (the Beffa form—see below) has a low frequency of 2 L-7 (and so it is more likely to be either 2 L-St/St or 2 L-St/7). Therefore identifications up to 1986 using 2 L-7 will be mostly (but not always) correct using the old diagnostic criteria of Vajime & Dunbar [5] if we also assume that heterozygotes are *S*. *soubrense*.

Surtees [19] and Surtees *et al*. [20] showed that *S*. *sanctipauli* in the montane forest east of the Volta lake was represented by a unique cytotype, the Djodji form of *S*. *sanctipauli*, characterised by inversion 1S-21. Subsequent entomological investigations showed that it was one of the most efficient vectors of onchocerciasis known to man [34] and that it was capable of extending its range northward during the rainy season from the montane forest into the savannah [35]. It was clearly a menace and in February–March 1988 the OCP extended larviciding operations to cover its entire dry-season breeding range, and the Djodji form of *S*. *sanctipauli* seemed to disappear and is now presumed extinct [36]. Our recent results (2006–2011) also failed to identify Djodji form from the Volta Region of Ghana (see Additional file 1). However, three specimens were identified as *S*. *sanctipauli* from a sample of larvae collected from Djodji 06-iii-2010 (along with seven *S*. *squamosum* and nine larvae which were unidentifiable due to the generally poor state of the material). These three specimens could not be sexed, but none of them were heterozygous for inversion 1S-21 (which is diagnostic of male Djodji form), and it is therefore possible that they were the Pra form rather than the Djodji form of *S*. *sanctipauli*. Djodji is 250–400 km downwind of the very large populations of the Pra form of *S*. *sanctipauli* which breeds in the river Pra and its tributaries [21].

There is no evidence that *S*. *sanctipauli* s.l. can migrate more than 150 km in one hop (although some other members of the *S*. *damnosum* complex can do so), but it has been recorded to increase its range over 230 km with the humid southwesterly winds when the inter-tropical convergence zone has passed overhead, and it is presumed that this is achieved by a stepwise spread northwards [35]. Therefore, these three specimens do not provide evidence for the survival of the Djodji form of *S*. *sanctipauli*, and it is noted that further specimens were not identified from three later samples from Djodji (in 2010 and 2011), or from any other site.

Meredith *et al*. [17] showed that populations of the *S*. *sanctipauli* subcomplex in Togo and Benin (east of the Volta lake) were a new cytotype, which they called the Beffa form, and it soon became clear that the Beffa form was a chromosomally and morphologically distinct geographic race within *S*. *soubrense*[16]. It was subsequently identified morphologically from the river Asukawkaw (=Asawkawkaw) in the Volta Region of Ghana [29]. Specimens of Beffa form have been identified chromosomally (see Additional file 1) using the diagnostic inversion 2S-6b from the Volta Region, and other specimens may be inferred as such because they were 2 L-7 heterozygotes or absent for 2 L-7 (see above). Beffa form has only been recorded from the Dayi river basin (rivers Agumatsu, Alavanyo, Dayi and Nubui) in three samples out of 146 (1976–2009), and those samples were prior to the elimination of Djodji form in 1988. Near the northern tip of the montane forest, in the rivers Kpaza, Koué and Wulubong, the Beffa form has been recorded in five samples (out of 68 samples, 1979–1996), all of which were prior to the elimination of the Djodji form. In the Asukawkaw basin, the Beffa and Djodji forms were both rare in the river Menu (each having been recorded in only one sample out of a total of 23 samples 1981–2010, and both prior to 1988), but more frequent in the rivers Asukawkaw and Wawa. In the river Asukawkaw, the Djodji form was recorded in 61 out of 99 samples (1975–Feb 1988) and 0 out of 10 samples (1989–2011), while in comparison, the Beffa form was recorded in 8 out of 99 samples (1975–Feb 1988) and 4 out of 10 samples (1989–2011), mostly from Asukawakaw Ferry. In the river Wawa (at Dodi Papase, Ahamansu and Djodji) the Djodji form was recorded in 85 out of 136 samples (1977–Feb 1988) and 0 (see above) out of 33 samples (June 1988–2011), while in comparison, the Beffa form was recorded in 17 out of 136 samples (1977–Feb 1988) and 1 out of 33 samples (June 1988–2011). In summary, the Beffa form has never been very common in the Volta region and there is no evidence that it has become more common since the elimination of the Djodji form.

In contrast, early evidence [37] indicated that *S*. *yahense* had increased its range since the elimination of the Djodji form in 1988. Fiasorgbor *et al*. [37] stated that up to 1988 east of the Volta lake in Ghana, *S*. *yahense* had been recorded in the river Agumatsu (at Wli Falls), the river Dayi (at Dzolu-Buem, Kudzra and Wegbe), Todzie (at Honuta and Kpedzie), and the river Tsatsadu (= river Alavanyo at Tsatsadu Falls), but after 1988 it was also identified from the river Asukawkaw (at Asukawkaw Bridge), the river Chai (at Kechebi), the river Menu (at Menusu), the river Wawa (at Djodji), and the river Wulubong (at Tukutukpene). Examination of Additional file 1 shows that *S*. *yahense* has continued to be found in the river Alavanyo (Tsatsadu Falls) (but there have been no new samples from the Wli Falls, Agumatsu river), the river Dayi (at Dayi Hotel, Kudzra and Wegbe, but there have been no new samples from Dzolu-Buem), and the Todzie river basin (including Angoe, Tale and Todzie rivers). However, analysis of all data since March 1988 (including new samples) does not support an increase in range, and if any expansion did occur, it has not continued.

*Simulium yahense* is generally rather uncommon downstream of Djodji on the river Wawa, and out of 40 samples from Ahamansu (1977–2010) and 31 samples from Dodi Papase (1977–2011), only one from each site was positive for *S*. *yahense* (dated 22-x-1996 in both cases), and these were only two of 18 samples taken since March 1988. *Simulium yahense* is also uncommon at Djodji, with only five out of 98 samples (1978–2011) positive, and three of these samples were taken before 1988 and only two out of 15 after March 1988. None of these data give any support for an extension of breeding by *S*. *yahense* into the river Wawa.

*Simulium yahense* was not identified from the river Menu before 1988 (16 samples, 1981–1987), but it was recorded in all five samples from 1989. However, this apparent extension of range may have been temporary, because it has not been recorded since 1989 (two samples, 2009–2010).

There have been 109 samples analysed from the river Asukawkaw of which ten were after March 1988. Only a single specimen of *S*. *yahense* has ever been identified (from Asukawakaw Bridge 12-v-1989, but not in new subsequent samples).

Our compilation of data indicates only one dated record of *S*. *yahense* from the river Wulubong and this was an uncertain identification, which pre-dated 1988 (07-vi-1981), although we could locate only a single sample after 1988 (22-x-1996). Similarly, we have only been able to find records of a single sample from the river Chai (Ketchebi 18-iv-1989) from which no *S*. *yahense* were identified, so we are unable to find any evidence for an extension of the range of *S*. *yahense* into this river. Furthermore, there is also no evidence that *S*. *yahense* has extended its range further north into the rivers Koue and Kpaza. There have been only three samples analysed since 1988, and all of them were negative for *S*. *yahense* (although a single specimen had been identified in 1980).

Wilson *et al*. [24] included the Volta Region of Ghana in their study of trends in the increasing frequency of savannah cytospecies (*S*. *damnosum* s.str. and *S*. *sirbanum*) in southern Ghana in relation to deforestation. We have compared the same database (updated with new samples since 1997 and some missing data added) to see if we can detect a continuation of this trend. We have no new samples from the rivers Koue, Kpaza and Wulubong, and in the river Asukawkaw there are no new samples from Dodofie and only one new sample from Asukawkaw Bridge, which was negative for savannah cytospecies, compared with 15/28 which were positive up to 1997. However, recent samples from Asukawkaw Ferry (2009–2011) gave strong evidence for an increase in savannah cytospecies because 6 out of 6 samples were positive in comparison with 4 out of 22 older samples (1981–1987) (Fisher Exact test, *P*-value = 0.001). There have been only two new samples from the river Wawa at Ahamansu, of which one contained a single larvae identified as *S*. *damnosum* s.str., and this is insufficient to look for trends. Savannah cytospecies were not identified from five new samples (2006–2011) from Dodi Papase, whereas 16 samples out of 26 were positive prior to 1997 (1977–1996). At Djodji, savannah flies were generally uncommon and were only found in a total of seven samples out of 98 (1978–2011). Of these, 5 of 83 were collected prior to March 1988 (and the elimination of the Djodji form), 2 of 8 were from April 1988–1997, and 0 of 7 were after 1997, yielding no evidence for a continuing trend of increasing frequency of savannah cytospecies in this area. Savannah flies were always very rare in the Todzie river basin (rivers Angoe, Tale and Todzie), with only one (uncertain) identification of a single *S*. *damnosum* s.str. from June 1982. However, we do not have any new samples to ascertain if this pattern has remained stable. Similarly, savannah flies have always been rare in the river Menu (only one positive sample out of 19, 1981–1989) and our two new samples (2009–2010) were negative. In the Dayi river basin, savannah flies have never been recorded from the smaller forested tributaries (river Alavanyo at Tsatsadu Falls and river Agumatsu at Wli Falls), and we have a single new sample from Tsatsadu Falls and this was also negative for savannah cytospecies. However, savannah cytospecies have been recorded in the river Dayi itself, and we have recent samples from Kudzra (one sample) and Wegbe (three samples). These samples do not, however, show a trend for increasing frequency of savannah flies (if anything they showed a slight drop in proportions of savannah cytospecies), but the numbers are too small for valid statistical inference.

## Discussion

After the closure of the OCP in 2002, and of the WHO special intervention zone (SIZ) in Ghana (around the area of Asubende), vector control operations are seldom implemented in the country and onchocerciasis control is reliant exclusively on regular mass delivery of ivermectin [38]. However, results to be presented elsewhere (but gathered within the same study period for the new samples reported here, 2009–2011) indicate that transmission is ongoing in some areas previously covered by the OCP as well as in areas not previously protected. Therefore, a thorough knowledge of the distribution of onchocerciasis vectors, and of any spatio-temporal changes that may have occurred, is crucial to the understanding of current risk and potential recrudescence of transmission, particularly in relation to the trends reported previously, of an expanding range of savannah cytospecies with increasing deforestation [24].

Cytotaxonomic identifications from 1,232 samples from southern Ghana (including 49 new and recent samples) were analysed to assess the distribution of cytospecies of the *Simulium damnosum* complex and any changes in distribution that may have happened during the period 1971–2011.

The rapids on the lower Volta river near Akosombo dam were subject to intermittent blackfly control (larviciding) by the Volta River Authority to reduce biting nuisance until 1981, when they were flooded by the new Kpong Dam. It is not clear whether the recrudescent population after each period of control was composed of immigrant flies or survivors of the preceding insecticide campaign, but the recrudescent blackfly populations were not always the same cytospecies as had occurred before control, strongly suggesting that on some occasions (at least) they were composed of immigrants. *Simulium damnosum* s.str. was usually present and is known to be a strongly migratory species [39], but *S*. *sirbanum* and the Beffa form of *S*. *soubrense* were found to be more sporadic in their presence. The Beffa form of *S*. *soubrense* is not such a strongly migratory species as *S*. *damnosum* s.str., and *S*. *sirbanum* is usually found further north than *S*. *damnosum* s.str. (so immigrant source populations may have been further away).

In the Volta Region (east of the Volta lake) the Djodji form of *S*. *sanctipauli* was eliminated by the OCP in 1988 and it has not been recorded since. Three recent specimens of *S*. *sanctipauli* were probably the Pra form of *S*. *sanctipauli* (not the Djodji form). There is no evidence that the Beffa form of *S*. *soubrense* has increased its occurrence in the Volta Region since 1988, and recent samples suggest that early evidence that *S*. *yahense* had increased its range since 1988 may have been the result of a temporary and short-lived expansion.

*Simulium yahense* is one of the most efficient onchocerciasis vectors within the *S*. *damnosum* complex amongst those that occurred in the study area, along with the Pra and Djodji forms of *S*. *sanctipauli* and the Beffa form of *S*. *soubrense*[25]. The eradication of the Djodji form does not seem to have resulted in a long-term increase in the range of either the Pra form of *S*. *sanctipauli*, the Beffa form of *S*. *soubrense* or *S*. *yahense*, and the other cytospecies present in the Volta Region are not particularly efficient vectors. This effect is likely to be beneficial to onchocerciasis control by CDTI, because the extinction of the Djodji form has removed one of the most efficient vectors in the area, and the other efficient vectors have not subsequently expanded their ranges to take its place.

Rivers flowing northwards and southwards from the forested Southern Voltaian Plateau show a strong zonation of their blackfly fauna, mostly with *S*. *squamosum* and *S*. *yahense* in the headwaters, sometimes with *S*. *damnosum* s.str. Further downstream, *S*. *sanctipauli* usually dominates in rivers flowing southwards (with a few savannah flies), and savannah cytospecies usually dominate in the rivers flowing northwards. We have no specific and direct evidence about whether this pattern has been stable over the years.

## Conclusions

It was previously shown that up to 1997 the savannah cytospecies (*S*. *damnosum* s.str. and *S*. *sirbanum*) were increasing in frequency in southern Ghana (probably as a result of deforestation) and our results indicate that this trend seems to be continuing in parts of the Asukawkaw river (in the Volta Region). However, throughout southern Ghana in general the number of recent samples (since 1997) has not been large, with consequently rather limited power to detect continuing changes in cytospecies composition. Deforestation has certainly continued since 1997, and if the trend in increasing frequency of savannah cytospecies is continuing in other rivers in southern Ghana, our analyses have been unable to detect it. This may be because of the limited number of samples (as mentioned above), or perhaps because our new samples mostly came from a few forested zones only, the Volta Region, the river Anum, and the lower section of the river Pra, which may not be representative of Ghana forests as a whole. Alternatively, it may be that savannah cytospecies have ceased to spread their geographical ranges after expanding during the 1975 to 1997 period.

Since onchocerciasis elimination has been recently documented in some (savannah) foci of Senegal, Mali and Nigeria that received annual or biannual ivermectin distribution without vector control [40,41], there is a new impetus for elimination of the infection reservoir where possible in Africa [14]. Knowledge of prevailing vector species and mix according to setting, their vectorial capacity, and seasonality patterns will be crucial to parameterise mathematical models of onchocerciasis transmission with which to investigate the feasibility of elimination in a number of scenarios [42]. In addition to cytotaxonomy data, we have collected data on the biting, parous, host feeding, and *O*. *volvulus* infection rates in a number of communities in southern Ghana within and outside the OCP area [43]. The implications of our findings for the transmission, control, and elimination of onchocerciasis in Ghana will be presented elsewhere.

## Abbreviations

APOC: African Programme for Onchocerciasis Control; CDTI: Community-directed treatment with ivermectin; OCP: Onchocerciasis Control Programme in West Africa; s.l.: sensu lato; s.str.: sensu stricto; WHO: World Health Organization.

## Competing interests

The authors declare that they have no competing interests.

## Authors’ contributions

M-GB, RAC, MDW & RJP conceived the project, which was designed by RJP, RAC, M-GB, MDW, DAB & PHLL Fieldwork was planned and carried out by RAC, PHLL, MDW, DAB, AT-K, MYO-A, M-GB & RJP Cytotaxonomy was carried out by RJP, JLC, DAB & MYO-A Historical data were gathered by RAC, RJP & LY Data interpretation was carried out by RJP, RAC, DAB & MYO-A. Manuscript was written by RJP with contributions from all authors. All authors read and approved the final version of the manuscript.

## Supplementary Material

Additional file 1 **Cytotaxonomic identifications of the *Simulium damnosum *complex from Ghana 1971–2011.** (In addition to references numbered as in the main text, this file refers to [44-48]). Click here for file

## Endnote

We wish to dedicate this paper to the memory of George Fiasorgbor in recognition of his considerable expertise in *Simulium damnosum* cytotaxonomy and the significant contribution he made to the success of the WHO Onchocerciasis Control Programme.
